# Chromatographic isolation of potentially novel antibiotic compounds produced by *Yimella sp.* RIT 621

**DOI:** 10.1186/s13104-023-06393-0

**Published:** 2023-06-22

**Authors:** Ian M. Freezman, Anutthaman Parthasarathy, Renata Rezende Miranda, Lizabeth M. Watts, André O. Hudson

**Affiliations:** 1 Thomas H. Gosnell School of Life Sciences, Rochester Institute of Technology, 85 Lomb Memorial Dr, 14623 Rochester, NY USA; 2 School of Chemistry and Biosciences, University of Bradford, BD7 1DP Bradford, UK

**Keywords:** *Yimella*, Antibiotics, Antibiotic resistance, Drug discovery

## Abstract

**Objective:**

Antibiotic resistant infections have become a global health crisis causing 1.2 million deaths worldwide in 2019 [1]. In a previous study, we identified a bacterium from a rare genus, *Yimella*, and found in an initial antibiotic screening that they produce broad-spectrum bactericidal compounds [2]. Herein, we focus on the characterization of these potential novel antimicrobial compounds produced by *Yimella* sp. RIT 621.

**Results:**

We used solid-phase extraction and C18 reverse-phase chromatography to isolate the antibiotic-active compounds found in organic extracts from liquid cultures of *Yimella sp*. RIT 621. We tracked the antimicrobial activity by testing the extracts in disc diffusion inhibitory assays and observed its increase after each purification stage.

## Introduction

Antibiotic resistance is a major problem facing the world of science and medicine. Each year in the United States, over two million individuals are infected with antibiotic resistant bacteria, and 23,000 people die as a direct result of these infections [3]. The use and overuse of antibiotics clinically and agriculturally has allowed microbes to exploit their genetic capacities to develop mechanisms of resistance, leading to resistant bacteria [4]. Since the 1980 and 1990 s, there has been an antibiotic discovery gap with no new class discovered since 1987. This lack of new antibiotics combined with the widespread use of agricultural and clinical antibiotics has led to the current resistance crisis [5].

In response, academics and small/medium sized companies have started antibiotic discovery programs on a significant scale. As of 2020, about 400 preclinical antibiotics projects were run by 314 institutions [6]. Over 660,000 bacterial genomes were deposited as of 2018 [7]. To find potential biosynthetic gene clusters (BGC) encoding novel natural products, genome mining is now routinely used. The combination of improved genome sequencing, BGC identification, and the arrival of new genetic tools such as CRISPR-Cas9 has propelled antibiotic prospecting into a new era [8].

Antibiotics have a variety of cellular targets including cell wall synthesis – the target of beta-lactam and glycopeptide antibiotics, protein synthesis – the target of tetracyclines and chloramphenicol, and DNA replication – the target of quinolones. These targets have known and observed resistance mechanisms manifesting in clinical infections, such as target modification, molecular bypass, and efflux [5]. This provides a rationale to find new antibiotics with more diverse mechanisms of action.

According to a recent study, less than 1% of bacteria and less than 5% of fungal species are currently known, out of which far fewer have been explored for bioactive compounds [9]. The explosion of genomics techniques led to the realization that microbes possess much greater potential to produce novel secondary metabolites than previously considered [10]. One of the cost-effective and low-tech ways to discover novel antibiotics is to sample more diverse microbes, rather than common sources such as Actinobacteria or Pseudomonas. Natural products from underrepresented bacterial species have greater potential in finding chemical novelty, and targeting these natural products allows for a greater potential of discovering antibiotics with more diverse mechanisms and more potent clinical potential [11].

The genus *Yimella* is made up of non-motile coccoid Gram-positive bacteria of the *Dermacocceae* family. There are limited studies on this genus, mainly pertaining to systematics, but few, if any, report on their secondary metabolism [2]. *Yimella sp.* RIT 621 was isolated in 2018 from a door handle on the campus of the Rochester Institute of Technology (RIT), and we discovered that it produces compounds that inhibit the growth of *Esherichia coli* and *Bacillus subtilis* [2]. The present study aimed to isolate and characterize these potentially novel antimicrobial natural products from *Yimella sp.* RIT 621 by applying different chromatographic techniques and tracking the active compounds at every purification stage.

## Main text

### Methods

#### Bacterial growth

*Yimella sp.* RIT 621 was plated from frozen glycerol stocks and grown in starter cultures of 5 mL of Tryptic Soy Broth (TSB) for 48 h at 30 ℃ at 100 rpm. The liquid culture was scaled-up to 100 mL followed by 1 L of TSB and grown under the same conditions. This process was repeated until a total of 10 L of liquid culture was produced.

#### Ethyl acetate extraction of culture medium

Scaled-up liquid cultures of *Yimella sp.* RIT 621 were centrifuged at 6,000 rpm for 20 min at 4 ℃, and the supernatant was decanted from the cell pellet. Sodium chloride was added to the supernatant, and the solution was acidified to pH < 2. Extractions were performed with 250 mL of ethyl acetate per 1 L of media, and the extracts were dried with anhydrous sodium sulfate. After filtration, the solvent was evaporated using a rotary evaporator, the resulting extract was resuspended in methanol, and the samples were then dried using a Speed-vac. A total of 8.385 g of crude extract was produced from 10 L of *Yimella sp.* RIT 621 liquid cultures. Blank extractions were also performed using uninoculated TSB media to serve as controls.

#### Sample preparation

The crude organic extract was resuspended into 10% methanol/water (v/v) solution and centrifuged for 20 min at 6,000 rpm at room temperature. The supernatant was decanted, evaporated using a Speed-vac, and resuspended into an appropriate volume of methanol in each case.

#### Mixed anion exchange chromatography

OASIS® MAX columns (Waters) were used for solid-phase extraction. The specific column parameters were 60 μm particle size, 80 Å pore size, 150 mg sorbent mass, and 6 mL total volume capacity (or column volume, CV). The extract crudes were dissolved in 10% methanol/water (v/v) to be loaded onto the columns. Six fractions were collected by eluting with increasing concentrations of methanol in water (25, 60, and 100% v/v), first as neutral solutions (fractions A1-3, respectively), then with the addition of 1% of formic acid (fractions A4-6, respectively). The fractions were then concentrated using a Speed-vac for use in bioassays and the following amounts were obtained of each fraction: A1 = 87 mg; A2 = 42 mg; A3 = 47 mg; A4 = 26 mg; A5 = 47 mg; A6 = 38 mg.

#### Disc-diffusion inhibitory bioassays

The assays were performed according to a previously reported protocol (Steiner et al. (2020). Briefly, *E. coli* was grown overnight in 20 mL of Luria-Bertani (LB) broth at 37 ℃ at 100 rpm. The culture was centrifuged for 20 min at 6,000 rpm. The cell pellet was resuspended in 20 mL of sterile phosphate buffered saline (PBS) pH 7.4, and 200 µL was mixed with 20 mL of warm LB agar. The mixture was plated in a square Petri plate, and 6 mm blank paper discs were overlaid equally spaced on the media. The standard inoculum consisted of a culture set at an OD_600_ of 0.1 before mixing with warm agar.

Crude extract or chromatography fractions dissolved in methanol were pipetted onto the discs (the exact amounts are indicated in the respective figure caption or on Table 1), and the plate was incubated at 30 ℃ for 16 h. The positive control consisted of 10 µL of 10 mg/mL of tetracycline. The negative control consisted of 40 µL of methanol. All plates were imaged using a Bio-Rad ChemiDoc MP imager, and the zones of inhibition (mm) around each disc were measured using a ruler.

**Table 1:** Comparative analysis of the samples used in the disc-diffusion inhibitory bioassays against *Escherichia coli* ATCC 25,922

Sample	Process Stage	ZOI (mm)	Mass Plated (mg)	Volume (µL)	ZOI/mg
Crude Extract	Extraction	14	20.49	30	0.683
A1	MAX	17	13.05	30	1.303
A2	MAX	11	6.3	30	1.746
A3	MAX	9	7.05	30	1.277

*These are the values obtained with one test (n = 1)

#### Reverse phase C18 fast protein liquid chromatography (FPLC)

The MAX chromatography fraction crude that showed antimicrobial activity was dissolved in methanol at a concentration of 100 mg/mL. The FPLC purification was performed using a Bio-Rad NGC Chromatography system equipped with a multiple-wavelength detector (λ = 215, 255, 280, and 495 nm) and an Agilent C18 column (ZORBAX Eclipse XDB 80Å C18, 9.4 × 250 mm, 5 μm). The analyses were carried out using acetonitrile with 0.1% formic acid (v/v) (= A) and purified water (= B) as the mobile phase, at a flow rate of 2.5 mL/min, in a gradient mode: 0–10 min at 0% B, 5 min gradient 0–10% B, 10 min at 15% B, 5 min gradient 15–20%B, 10 min at 20% B, 5 min gradient 20–30% B, 10 min at 30% B. The resulting chromatograms were analyzed using the ChromLab program provided by the manufacturer.

## Results and discussion

**Fig. 1:**
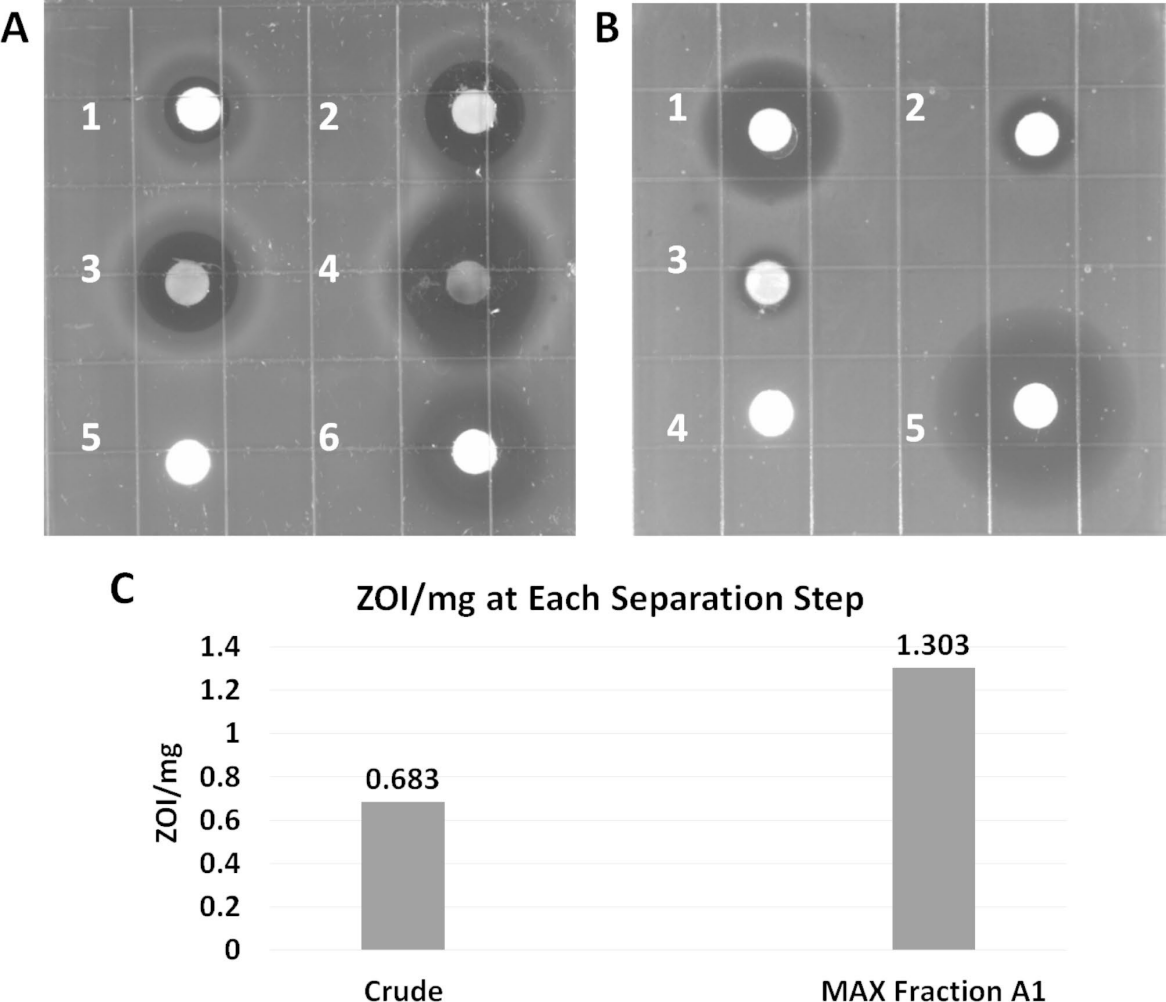
*Yimella* sp. RIT 621 produces antibiotic activity against *Escherichia coli* ATCC 25,922. (**A**) Disc-diffusion inhibitory assay with increasing volumes/amounts of a 683 mg/mL solution of *Yimella* sp. RIT 621 spent TSB medium extract applied to discs 1–4: 10 µL, 6.83 mg (1); 20 µL, 13.66 mg (2); 30 µL, 20.49 mg (3); 40 µL, 27.32 mg (4); 40 µL of methanol (5) and 10 µL, 0.1 mg of tetracycline (6) were used as negative and positive controls, respectively. (**B**) Disc-diffusion inhibitory assay with MAX-collected fractions: A1 (1), A2 (2), A3 (3), methanol (4), 10 mg/mL tetracycline solution; volumes: 30 µL on discs 1–4, and 10 µL on disc 5. The respective amounts can be found on Table 1. (**C**) Comparison between the zone of inhibition (ZOI, mm) per mass (mg) plated of the extract crude and MAX fraction A1 – the MAX fraction showed greater activity then the extract crude, indicating that this separation method was effective in isolating the antibiotic-active compounds produced by *Yimella* sp. RIT 621 from the non-active substances

Ethyl acetate spent TSB medium extracts of *Yimella* sp. RIT 621 were tested for antimicrobial activity against *Escherichia coli* (reference strain ATCC 25,922) in a disc diffusion inhibitory assay (Fig. 1A; Table 1). Increasing amounts of the crude extract were applied to sterile discs equally spread-out on an agar plate inoculated with the bacteria and the zones of inhibition (ZOIs) around each disc were measured. The ZOI values increased with the amount of extract, showing a dose-dependent susceptibility of the *E. coli* bacteria towards the *Yimella* sp. RIT 621 spent medium extracts.

The crude extracts were then subjected to separation via solid-phase MAX chromatography. The antibiotic activity was tracked by testing each collected fraction in another disc diffusion inhibitory assay following a similar procedure as mentioned above (Fig. 1B; Table 1). Among the collected fractions, fraction A1 (25% methanol in water, v/v) showed the highest bacterial growth inhibition followed by fractions A2 and A3, which had increased amounts of methanol (60 and 100% v/v, respectively), suggesting that the antibiotic-active compounds exhibit greater polarity than the non-active ones. Fractions A4-6 did not show any activity (data not shown). Moreover, fraction A1 showed roughly twice the activity against *E. coli* when compared to the crude extract (Fig. 1C; Table 1), indicating that the MAX method was effective in isolating the active compounds from other substances present in the bacterial crude extract.

**Fig. 2:**
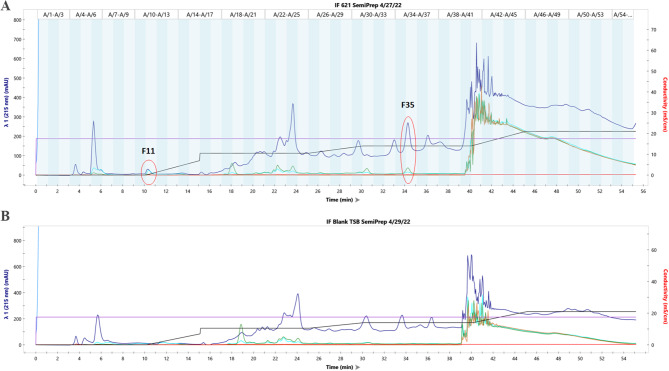
Semi-preparative C18 reverse-phase FPLC was performed with a MAX fraction A1 to further separate and isolate the antibiotic compound(s). The chromatogram of the active sample (**A**) shows two peaks that stand out (fractions 11 and 35) in contrast to the blank sample chromatogram (**B**). Both samples were injected as 100 mg/mL solutions in methanol. The traces are: λ1 = 215 nm (navy), λ2 = 255 nm (teal), λ3 = 280 nm (green), λ4 = 495 nm (brown), conductivity (red), % B (black), system pressure (blue), and flow rate (violet)

Based on these results, fraction A1 was selected for further purification and analysis through liquid chromatography, and the resulting chromatogram can be seen in Fig. 2. A 100 mg/mL solution of the active sample in methanol was used in the separation (Fig. 2A). A sample consisting of an MAX A1 fraction after separation of a blank TSB medium crude extract (no bacteria) was used as a negative control for chromatogram comparison (Fig. 2B). Two main peaks stand out in the active sample with retention times of approximately 10 and 34 min (fractions 11 and 35, respectively). These peaks are not found in the blank chromatogram and likely correspond to unique compounds produced by *Yimella* sp. RIT 621. The presence of unique compounds in the chromatogram from the active sample highly suggests that secondary metabolites produced by *Yimella* sp. RIT 621 are the ones causing the observed growth inhibition of *E. coli* in the bioassays. Additionally, the genome of *Yimella* sp. RIT 621 has been previously sequenced and analyzed for such compounds using antiSMASH [6], and four gene clusters potentially encoding pathways for the synthesis of secondary metabolites were found. Future work is needed to characterize the antibiotic-active compounds produced by this bacterium, including scaling-up the FPLC separations to collect a greater amount of the unique fractions found in the active sample, testing these fractions in bioassays to confirm their antimicrobial activity, and further spetrometrical analysis to identify the chemical structures of the novel antibiotic compounds produced by *Yimella* sp. RIT 621.

### Limitations

Minimum inhibitory concentration (MIC) values and structural properties of the antibiotic compounds are still unknown.

## Data Availability

This whole-genome project for *Yimella* sp. RIT 621 has been deposited in GenBank under the accession number SEIP00000000.
